# Endothelial Atypical Receptors for Chemoattractants in Lung Immune Surveillance

**DOI:** 10.1002/eji.70259

**Published:** 2026-08-17

**Authors:** Mattia Laffranchi, Eleonora Bonanni, Francesca Sozio, Miriam Turchetti, Giuseppe Sciumè, Marcus Thelen, Annalisa Del Prete, Raffaella Bonecchi, Silvano Sozzani

**Affiliations:** ^1^ Department of Molecular Medicine Sapienza University of Rome Rome Italy; ^2^ Istituto Pasteur‐Fondazione Cenci Bolognetti Rome Italy; ^3^ Institute for Research in Biomedicine Università della Svizzera Italiana Bellinzona Switzerland; ^4^ Department of Molecular and Translational Medicine University of Brescia Brescia Italy; ^5^ Department of Immunology IRCCS Humanitas Research Hospital Milan Italy; ^6^ Department of Biomedical Sciences Humanitas University Milan Italy

**Keywords:** cell migration, chemoattractant receptors, chemokine receptor, chemokine, endothelium, immune system, immunology, inflammation, lung cancer, lung

## Abstract

Chemokines and their receptors play a pivotal role in the initiation and regulation of inflammation through the orchestration of leukocyte extravasation and directed migration toward sites of tissue injury. Tight control of chemokine gradients within tissues is essential to ensure that inflammatory responses remain transient and properly resolved. When this regulatory mechanism fails, dysregulated chemokine signaling can contribute to the development of chronic inflammation. Atypical receptors for chemoattractants comprise atypical chemokine receptors (ACKRs) and the chemerin‐presenting receptor CCRL2. ACKRs perform specialized functions enabling the fine‐tuning of chemokine gradients, primarily through the scavenging, sequestration, or redistribution of chemokines. By regulating their spatial and temporal availability, ACKRs play a key role in limiting excessive leukocyte recruitment and promoting inflammation resolution. The lungs are in a dynamic equilibrium between immune activation and homeostasis. Rapid and tightly regulated immune cell recruitment is essential for effective host defense while preventing tissue damage. In this context, ACKRs expressed by specialized lung endothelial cells are emerging as critical regulators of leukocyte trafficking and inflammatory resolution. Given the paucity of studies in this area, this review summarizes current knowledge of ACKRs and CCRL2 in lung immune surveillance and discusses their potential as therapeutic targets in lung diseases.

## Graphical abstract

Control of chemokine gradients within tissues is achieved by atypical chemokine receptors (ACKRs). The lungs require tightly regulated leukocyte recruitment for effective host defense while preventing tissue damage. In this context, ACKRs expressed by lung endothelial cells are emerging as critical regulators of leukocyte trafficking and inflammatory resolution.

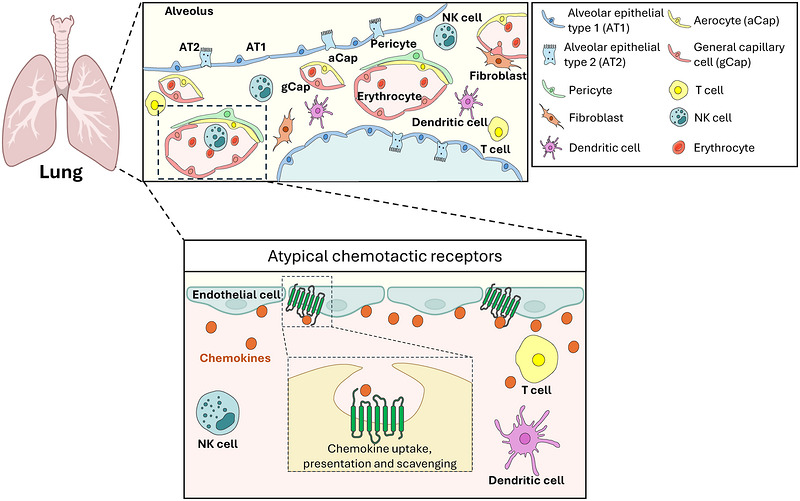

## 1 Introduction

The coordination of immune responses within tissues relies on the precise regulation of leukocyte trafficking, a process that is tightly controlled to ensure effective host defense while preventing excessive inflammation [1, 2, 3, 4, 5, 6, 7]. In barrier organs such as the lung, this balance is particularly critical, as continuous exposure to environmental stimuli requires rapid immune activation coupled with efficient resolution mechanisms. Failure to maintain this equilibrium can result in chronic inflammation and tissue damage.

The lung represents a highly specialized immunological environment, where structural and cellular organization contributes to the fine control of immune cell recruitment. In particular, the vascular compartment plays a central role in orchestrating leukocyte entry into the tissue. Endothelial cells (ECs), positioned at the interface between circulation and parenchyma, actively regulate immune cell extravasation and distribution, thereby shaping local inflammatory responses [8, 9].

In recent years, atypical chemokine receptors (ACKRs) have emerged as key regulators of chemokine‐driven processes [2, 10, 11]. Unlike canonical chemokine receptors, ACKRs do not couple to heterotrimeric G proteins and therefore do not initiate G protein‐dependent signaling. Instead, they can recruit β‐arrestins, which promote receptor internalization and, for most ACKRs, ligand scavenging [2]. In ECs, the importance of chemokine‐induced β‐arrestins recruitment to ACKRs for intracellular signaling remains to be determined. CCRL2 shares the inability to activate G protein‐dependent signaling but differs from bona fide ACKRs in that it neither efficiently recruits β‐arrestins nor undergoes ligand‐induced internalization. Moreover, among the ACKR family, ACKR1 is unique in its ability to transport and display intact chemokines on the luminal surface of endothelial cells [12, 13]. Similarly, endothelial CCRL2 can present its ligand chemerin to circulating leukocytes expressing ChemerinR1 (CMKLR1) [14]. Through their ability to bind multiple chemokines, often with broad ligand promiscuity, and in some cases, non‐chemokine ligands, ACKRs play a central role in regulating the availability, distribution, and biological activity of chemoattractants within tissues (Tables 1 and 2)

**TABLE 1:** Recognized ligands for the atypical receptors for chemoattractants.

Receptor	Chemokine families	Number of validated chemokine ligands	Non‐chemokine ligands	References
ACKR1	CC + CXC	22	LukE (*S. aureus*), HlgAB (*S. aureus*), DBP (*P. vivax*, *P. knowlesi*)	[69, 70, 71, 72, 73, 74, 75]
ACKR2	CC + CXC	19	gp120 (HIV), Staphopain A (*S. aureus*)	[76, 77, 78, 79, 80, 81, 82, 83, 84]
ACKR3	CXC	2	vCCL2 (HHV‐8), MIF, opioid peptides	[85, 86, 87, 88, 89, 90]
ACKR4	CC	5	None	[31, 91, 92, 93]
ACKR5	CC + CXC + XCL + CX3CL	42	Opioid peptides, apelin, PACAP, lipoproteins	[46, 47, 58, 94, 95]
CCRL2	None	0	Chemerin	[96, 97]

*Note*: List of validated ligands for the atypical receptors for chemoattractants divided for chemokine family and non‐chemokine targets.

Abbreviations: DPB: Duffy binding protein, HlgAB: gamma‐hemolysin component AB, LukE: leucotoxin LukE; *P. knowlesi*: *Plasmodium knowlesi*, *P. vivax*: *Plasmodium vivax*; *S. aureus*: *Staphylococcus aureus*.

**TABLE 2:** Promiscuity and overlaps of recognized chemokines by the atypical chemokine receptor family members.

Chemokine	Chemokine receptor	ACKR	Reference
CXCL1	CXCR2	ACKR1 (H&M, +++), ACKR2 (M, +), ACKR5 (H, +)	[69, 76, 94]
CXCL2	CXCR2	ACKR1 (H&M, +++), ACKR2 (H, +), ACKR5 (H, ++)	[46, 70, 76, 95]
CXCL3	CXCR2	ACKR1 (H&M, +++), ACKR5 (H, ++)	[58, 69]
CXCL4	CXCR3, CCR1	ACKR5 (H, +++)	[94]
CXCL5	CXCR2	ACKR1 (H&M, +++), ACKR2 (H&M, +), ACKR5 (H, ++)	[47, 70, 76]
CXCL6	CXCR1, CXCR2	ACKR1 (H&M, +++), ACKR5 (H, +)	[58, 70]
CXCL7	CXCR1, CXCR2	ACKR5 (H, +)	[94]
CXCL8	CXCR1, CXCR2	ACKR1 (H&M, +++), ACKR5 (H, ++)	[69, 70, 94]
CXCL9	CXCR3	ACKR1 (H&M, +), ACKR5 (H&M, ++)	[46, 70, 95]
CXCL10	CXCR3	ACKR1 (H&M, +), ACKR2 (H&M, +), ACKR5 (H&M, +++)	[46, 69, 70, 77, 95]
CXCL11	CXCR3	ACKR1 (H&M, +++), ACKR2 (H&M, +), ACKR3 (H&M, +++), ACKR5 (H&M, +++)	[46, 70, 76, 85, 95]
CXCL12	CXCR4	ACKR1 (H&M, dimer, +++), ACKR2 (H&M, +), ACKR3 (H&M, +++), ACKR5 (H&M, +++)	[46, 71, 72, 76, 85, 95]
CXCL13	CXCR5	ACKR1 (H&M, +), ACKR5 (H&M, +++)	[70, 94]
CXCL14	CXCR4	ACKR5 (H&M, +++)	[94]
CXCL16	CXCR6	ACKR5 (H&M, +)	[46, 95]
CXCL17	GPR25	ACKR5 (H&M, +++)	[58]
CCL1	CCR8	ACKR1 (H&M, +), ACKR5 (H, +++)	[58, 70]
CCL2	CCR2	ACKR1 (H&M, +++), ACKR2 (H&M, +++), ACKR5 (H, +++)	[58, 69, 78]
CCL3	CCR1, CCR5	ACKR2 (H&M, +), ACKR5 (H +)	[58, 79]
CCL3L1	CCR1, CCR3, CCR5	ACKR2 (H, +++)	[78, 79]
CCL4	CCR5	ACKR2 (H&M, +++), ACKR5 (H, +++)	[58, 80]
CCL5	CCR1, CCR3, CCR5	ACKR1 (H&M, +++), ACKR2 (H&M, +++), ACKR5 (H, ++)	[46, 69, 70, 78, 79, 95]
CCL7	CCR1, CCR2, CCR3	ACKR1 (H&M, +++), ACKR2 (H&M, +++)	[70, 81]
CCL8	CCR1, CCR2, CCR3	ACKR1 (H&M, +), ACKR2 (H, +++), ACKR5 (H, ++)	[58, 70, 78, 79]
CCL11	CCR3, CCR5	ACKR1 (H&M, +++), ACKR2 (H&M, +++), ACKR5 (H&M, +)	[58, 78, 79]
CCL12	CCR2	ACKR2 (M, +++)	[78]
CCL13	CCR2, CCR3	ACKR1 (H&M, +++), ACKR2 (H, +++), ACKR5 (H, +++)	[47, 80, 82]
CCL14	CCR1, CCR3, CCR5	ACKR1 (H&M, +++), ACKR2 (H, +++), ACKR5 (H, +++)	[47, 80]
CCL15	CCR1, CCR3	ACKR5 (H, ++)	[94]
CCL16	CCR1, CCR2, CCR5, CCR8	ACKR1 (H&M, +++), ACKR5 (H, +++)	[70, 94]
CCL17	CCR4	ACKR1 (H&M, +++), ACKR2 (H&M, +++), ACKR5 (H&M, +)	[70, 83, 94]
CCL18	CCR8	ACKR1 (H&M, +), ACKR5 (H, +)	[70, 94]
CCL19	CCR7	ACKR4 (H&M, +++), ACKR5 (H, +++)	[31, 91, 94]
CCL20	CCR6	ACKR4 (H&M, ++), ACKR5 (H, ++)	[92, 93, 94]
CCL21	CCR7	ACKR4 (H&M, +++), ACKR5 (H, ++)	[31, 91, 95]
CCL22	CCR4	ACKR2 (H&M, +++), ACKR4 (H&M, ++), ACKR5 (H&M, ++)	[83, 92, 93, 94]
CCL23	CCR1	ACKR5 (M, ++)	[94]
CCL24	CCR3	ACKR5 (H&M, +++)	[94]
CCL25	CCR9	ACKR4 (H&M, +++), ACKR5 (H&M, +++)	[31, 91, 94]
CCL26	CCR3	ACKR5 (H, ++)	[94]
CCL27	CCR3	ACKR5 (H&M, +++)	[94]
CCL28	CCR10	ACKR5 (H&M, +++)	[94]
CX3CL1	CX3CR1	ACKR5 (H, +)	[94]
XCL1	XCR1	ACKR5 (H, ++)	[94]
XCL2	XCR1	ACKR5 (H, +)	[94]

*Note*: Promiscuity of atypical chemokine receptors summarized by the recognition of targeted chemokines. H: human; M: mouse. + weak, ++ medium and +++ strong ligand‐receptor interaction, respectively.

Through these functions, they contribute to the spatial organization of immune responses and the prevention of excessive leukocyte accumulation [2, 15, 16, 17, 18].

Within the lung, atypical chemoattractant receptors are expressed by specialized endothelial subsets and are increasingly recognized as key regulators of immune surveillance and inflammatory resolution [19, 20]. However, despite growing interest in their biology, their specific roles in the pulmonary vasculature and their contribution to disease remain incompletely understood.

In this review, we summarize current knowledge on the role of endothelial atypical receptors for chemoattractants in lung immune regulation, discuss their involvement in inflammatory and pathological conditions, and explore their potential as therapeutic targets.

### 1.1 The Role of ACKRs in Lung Inflammation

The lung, to ensure efficient gas exchange, is characterized by a highly vascularized alveolar compartment, composed of a specialized endothelial cell network. Similar to the alveolar epithelium, comprising alveolar type 1 (AT1) and type 2 (AT2) cells, the alveolar capillary endothelium is also heterogeneous and can be divided into aerocyte (aCap) and general capillary (gCap) ECs. aCaps are primarily optimized for gas exchange and immune cell recruitment, while the gCaps regulate vasomotor tone, antigen presentation, immunomodulation, and also serve as a reservoir of endothelial progenitor cells during tissue repair [21, 22, 23, 24].

Owing to its peculiar vascular structure, the lung employs distinct mechanisms for leukocyte extravasation. The alveolar capillary network represents the predominant endothelial surface of the lung and the principal site of neutrophil recruitment during acute alveolar inflammation. Of note, leukocyte recruitment in alveolar capillaries differs from the classical venular paradigm, relying largely on mechanical sequestration [25] and exhibiting variable dependence on selectins and β2 integrins depending on the inflammatory stimulus [26, 27]. In contrast, leukocyte recruitment in bronchial and peribronchial tissues largely follows the classical rolling, adhesion, and transmigration cascade occurring in postcapillary venules [8]. In addition, leukocyte transmigration has been reported in pulmonary arterioles under severe inflammatory conditions [28]. However, several important questions remain unresolved, including whether transmigration occurs predominantly via paracellular or transcellular routes, whether gCap and aCap endothelial cells differentially support transendothelial migration (TEM), also considering that aCaps constitutively express *Icam1*, whereas gCaps upregulate the expression of adhesion molecules only during inflammation [21, 26]. Similarly, the roles of junctional molecules (e.g., JAM family proteins) and endothelial glycocalyx glycosaminoglycans (GAGs) in regulating this process are still poorly understood. Nevertheless, both aCaps and gCaps contribute to immune cell extravasation, and their selective expression of atypical chemoattractant receptors may locally modulate chemotactic gradients and shape leukocyte recruitment. Following transmigration, leukocytes transiently localize within the thin interstitial space before crossing the epithelial layer to reach the alveolar space [6, 17, 25].

**FIGURE 1:**
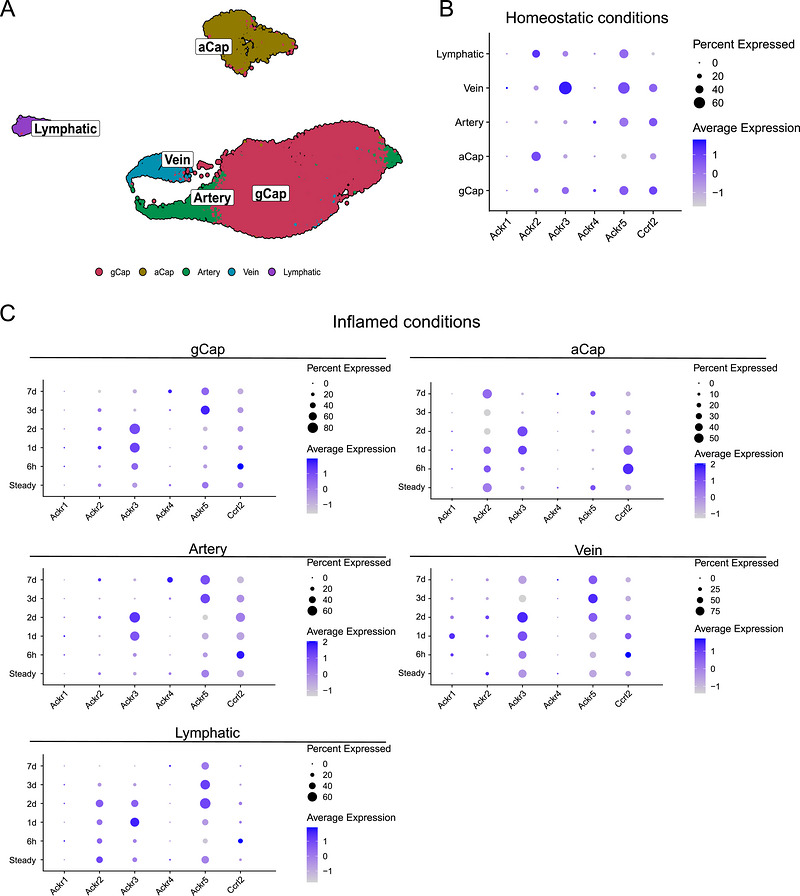
The expression of atypical chemoattractant receptors in the lung vasculature at steady state or during acute inflammation. (A) Umap representing a scRNA‐seq dataset of mouse lung endothelial cells [26]. The data were downloaded from GSE148499 and explored with Seurat v5.2 [98]. (B) Dot plot presenting the normalized expression of atypical chemoattractant receptors in the lung endothelium at homeostasis. (C) Dot plot presenting the normalized expression of atypical chemoattractant receptors, divided by population, as a time course upon intraperitoneal LPS injection (for more details, please refer to Zhang et al. [26]).

Continuous exposure to inhaled particles and pathogens places the lung in a dynamic balance between immune activation and homeostasis. To avoid excessive or chronic inflammation, lungs evolved complex immunoregulatory mechanisms that spatially confine immune responses. At steady state, the expression of ACKRs is tightly controlled in a cell type‐specific manner, as revealed by scRNA sequencing analysis of murine lung ECs [26] (Figure 1A,B). This spatial regulation of chemokines enables selective regulation of leukocyte subsets, thereby supporting rapid response to stressors and pathogens, while limiting tissue damage.

During inflammation, atypical chemoattractant receptor expression is dynamically regulated. In a model of LPS‐induced systemic inflammation, both aCaps and gCaps in the lungs display time‐ and cell type–specific changes in the expression of *Ackr2*, *Ackr3*, *Ackr5 (Gpr182)* and *Ccrl2* (Figure 1C) [26, 27]. In addition, *Ackr1*, expressed in the endothelial compartment, may contribute to chemokine gradient formation through transcytosis and display of chemokines on the endothelial surface [17, 24, 29]. Of note, only a few reports have documented Ackr4 expression in the lung endothelial compartment [30, 31]. Collectively, the coordinated and cell‐type‐specific expression of atypical chemoattractant receptors contributes to shaping the local inflammatory microenvironment and promoting resolution of inflammation (Figure 1C) [21, 26].

Due to the central role of lung ECs in immune regulation, their dysfunction is associated with multiple acute and chronic multifactorial diseases, including acute respiratory distress syndrome (ARDS), chronic obstructive pulmonary disease (COPD) and pulmonary hypertension (PH) [32]. However, the specific contribution of endothelial atypical chemoattractant receptors to these pathologies remains incompletely understood.

In patients and in pulmonary fibrosis models, ACKR1^+^ ECs are frequently observed in proximity to immune and alpha‐smooth muscle actin positive (αSMA^+^) mesenchymal cells forming niches that promote fibroblast activation and disease progression [33, 34]. Consistent with this, *Ackr1*‐ko mice are protected from neutrophil‐mediated lung injury, suggesting a pathogenic role linked to chemokine regulation [35, 36]. Data generated with full *Ackr1*‐ko mice should be interpreted with caution, as ACKR1 is expressed by both ECs and erythrocytes. Consequently, the relative contribution of endothelial cells versus erythrocyte ACKR1 cannot be readily distinguished. Nevertheless, ACKR1 expressed on erythrocytes is considered to act as a sink for chemokines, controlling chemokine plasma levels [13, 37], whereas ACKR1 on ECs can act as a scavenger, transcytosing them to the luminal surface, enhancing leukocyte migration [38]. In addition, in postcapillary venules, ACKR1 regulates chemokine availability by both degrading and transporting them from the abluminal to the luminal endothelial surface, where they can contribute to the recruitment of neutrophils and other immune cells [14].

ACKR2, expressed by lymphatic and aCap ECs (Figure 1B) [20, 39], functions as a scavenger receptor mainly for inflammatory CC chemokines and plays a key role in regulating leukocyte trafficking (Tables 1 and 2). In the lung, however, its role appears context‐dependent. In several models of inflammation, *Ackr2* deficiency is associated with reduced tissue injury, decreased leukocyte infiltration, and improved lung function [40]. In general, altered chemokine availability influences the recruitment of CCR2^+^ and CCR5^+^ IFNγ‐producing γδ T cells and modulates the Th17 response, which is critical for fibrosis progression. Similarly, in influenza A virus infection, *Ackr2* ko displays increased recruitment of CCR5^+^ lymphocytes, improved viral clearance, and reduced lung damage [41]. Additional evidence suggests that ACKR2 may also control T lymphocyte trafficking, highlighting a vascular compartment‐specific role in controlling immune cell entry into the lung [20]. These findings suggest that ACKR2, despite its canonical anti‐inflammatory function, may contribute to disease progression by reshaping chemokine gradients in specific contexts.

ACKR3 (CXCR7), expressed in both venous and capillary compartments (Figure 1) [42], also exhibits context‐dependent functions. In acute injury models, such as intratracheal injection of bleomycin or hydrochloric acid (HCl), ACKR3 retards fibrosis; its expression appears protective, limiting fibrosis progression. Whereas chronic injury leads to its downregulation and the emergence of a macrophage‐driven pro‐fibrotic environment [43]. In patients with interstitial pulmonary fibrosis, ACKR3 expression is reduced, further supporting its role in tissue homeostasis [43]. However, in LPS‐induced acute lung injury, ACKR3 antagonism improved lung recovery via increased serum levels of CXCL11 and CXCL12, highlighting the complexity of its function [44].

Evidence for other ACKRs remains limited. For example, ACKR4‐mediated scavenging of CCL21 has been implicated in regulating CCR7‐dependent dendritic cell (DC) trafficking, but data in the lung context are still scarce [30, 45]. Similarly, ACKR5, the most recently identified member of the ACKR family and characterized by a broad chemokine scavenging capacity (Tables 1 and 2) [10, 46], is expressed in vascular ECs of the lung (Figure 1) [47]; however, its role in lung inflammation responses has not yet been identified.

Overall, current evidence on the roles of ACKR expression in lung ECs remains limited. Further studies are required to define their contribution to immune surveillance and to evaluate their potential as therapeutic targets in pulmonary diseases.

### 1.2 ACKRs and CCRL2 in Lung Cancer and Immune Surveillance

**FIGURE 2:**
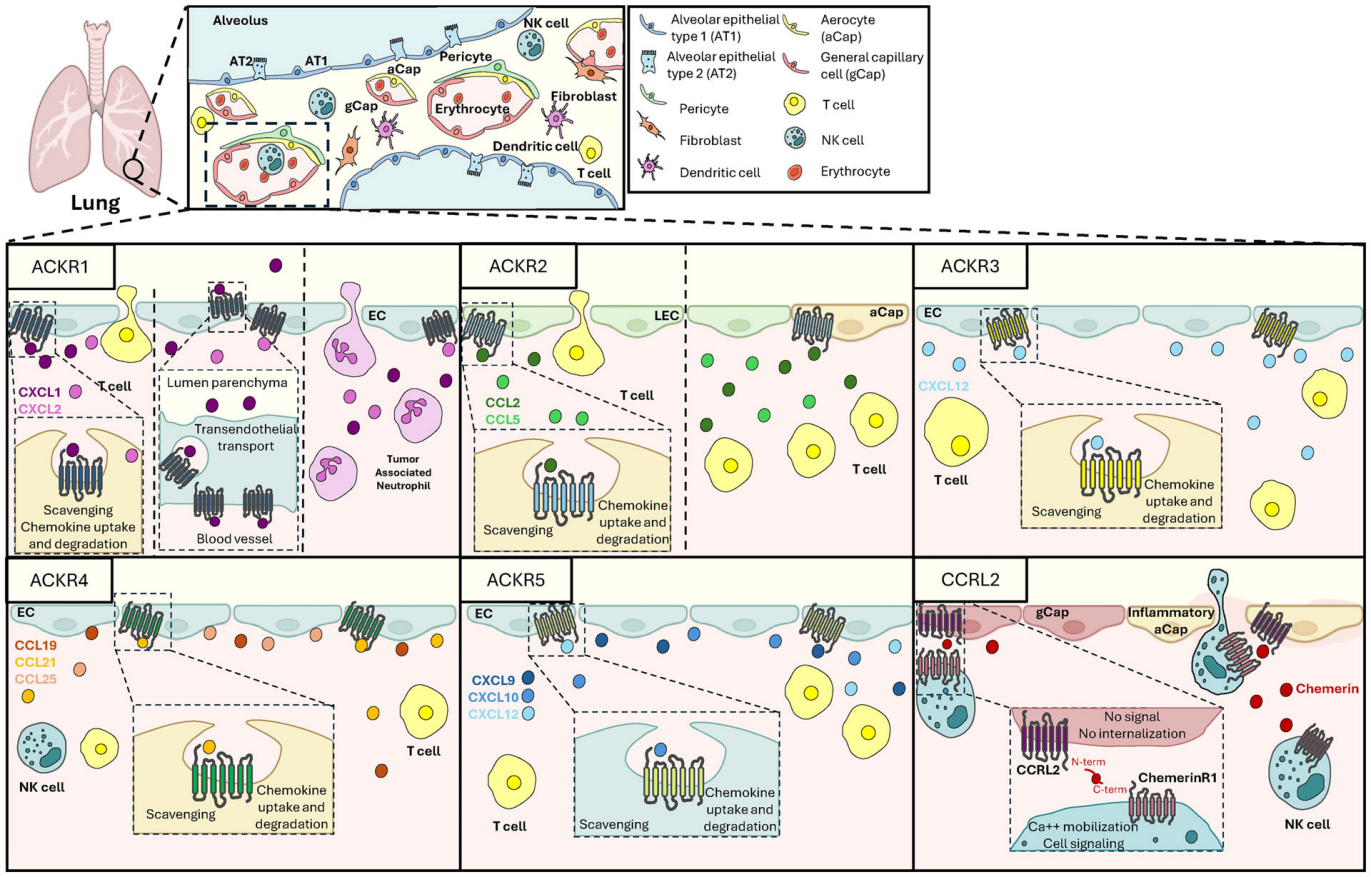
The role of atypical chemoattractant receptors in modulating cancer immune responses. ACKR1 expression by lung endothelial cells can promote infiltration of cytotoxic lymphocytes, supporting antitumor immunity. Conversely, in premetastatic niches, endothelial ACKR1 promotes neutrophil recruitment and metastatic colonization by regulating chemokine availability through transendothelial chemokine transport and presentation. ACKR2 in primary tumors is protective as it limits the accumulation of pro‐tumoral myeloid cells by reducing chemokine‐driven inflammation. Meanwhile, in metastatic niches, ACKR2 activity has been linked to tumor progression. ACKR3 expression enhances tumoral vascular permeability, tumor cell extravasation, and the establishment of a supportive metastatic niche by modulating the CXCL12/CXCR4 axis; ACKR4 expression promotes lung metastasis mainly by modulating NK cell–dependent responses. ACKR5 expression in lymphatic endothelial cells from melanoma models indicates that it has pro‐tumoral roles by restricting T cell infiltration. CCRL2 expressed by gCap or inflammatory aCaps acts as a chemerin‐presenting molecule by presenting chemerin to innate immune cells positive for the conventional chemerin receptor ChemerinR1.

Chemokines are known to regulate the composition of the immune tumor microenvironment (TME) [1, 5, 48]. Similar to inflammatory conditions, ACKRs expressed by lung endothelial and stromal cells within the TME exert complex and context‐dependent functions by modulating chemokine gradients (Figure 2). Their tightly regulated expression can lead to divergent biological outcomes depending on tissue and disease stage. In primary tumors, ACKRs are often associated with protective effects, largely due to their ability to shape chemokine gradients and limit the recruitment of immunosuppressive myeloid populations [37, 49, 50]. However, this paradigm does not fully extend to metastatic sites such as the lung. In this organ, endothelial ACKR expression, physiologically involved in maintaining tissue homeostasis, may instead facilitate metastatic seeding and growth.

ACKR1 exemplifies this duality. In primary tumors, endothelial ACKR1 expression correlates with increased infiltration of cytotoxic T lymphocytes, thereby supporting antitumor immunity [51]. In contrast, within the lung premetastatic niche, tumor‐derived factors induce ACKR1 upregulation on ECs, promoting neutrophil recruitment and creating a permissive environment for metastatic colonization [52].

ACKR2 similarly displays a context‐dependent role. In primary tumors, ACKR2 limits the accumulation of pro‐tumoral myeloid cells by dampening chemokine‐driven inflammation [53]. Conversely, in metastatic settings, including melanoma and breast cancer, ACKR2 activity has been associated with tumor progression [54, 55]. Mechanistically, its chemokine‐scavenging function may reduce the recruitment of effector T cells in the lung. Consistent with this, endothelial‐selective deletion of ACKR2 enhances T cell infiltration and activation, ultimately restricting metastatic outgrowth [20].

ACKR3 adds further complexity due to its dual function as both a scavenger receptor and an activator molecule via β‐arrestin‐dependent intracellular pathways that still need extensive characterization [37]. Nonetheless, in the lung endothelium, ACKR3 is upregulated in response to tumor‐derived signals and promotes metastasis [48]. Here, the predominant effect of ACKR3 is the scavenging of CXCL12, thereby reshaping gradients mediated by the CXCL12/CXCR4 axis that regulate tumor cell retention at primary sites, endothelial activation, and leukocyte trafficking. Through these combined effects, ACKR3 may enhance vascular permeability, tumor cell extravasation, and the establishment of a supportive metastatic niche. Although ACKR3 efficiently scavenges both CXCL12 and CXCL11, the biological consequences of CXCL11 scavenging remain largely unexplored. While extensive evidence demonstrates that ACKR3 regulates tumor progression and inflammation through modulation of the CXCL12/CXCR4 axis, whether ACKR3 limits CXCR3‐dependent recruitment of effector CD8^+^ T cells, Th1 cells, and NK cells by scavenging CXCL11 has received remarkably little experimental attention [56].

ACKR4, a scavenger of CC chemokines (Tables 1 and 2), also plays a critical role in immune cell positioning. In the lung, stromal ACKR4 expression promotes metastasis across multiple tumor types, including B16 melanoma, 3LL lung carcinoma, and RM1 prostate carcinoma [57]. ACKR4 alters chemokine availability within the tissue, thereby affecting the localization and function of immune cells. By modulating the availability of the CCR7 ligand CCL21, the genetic deletion of ACKR4 enhances the recruitment and activation of CD103+ DCs and CD8+ T cells resulting in improved anti‐tumor immunity [57]. This highlights the importance of chemokine gradient regulation in controlling immune surveillance.

Although data on ACKR5 in lung ECs during cancer progression remain limited, evidence from melanoma models indicates that ACKR5 expression in lymphatic endothelial cells restricts T cell infiltration. Genetic deletion of *Ackr5* increases intratumoral CXCL9 and CXCL10 levels, enhancing CXCR3‐dependent T cell recruitment and promoting antitumor immunity, including improved responsiveness to immune checkpoint blockade and adoptive cell therapies [58].

Beyond ACKRs, other atypical receptors for chemoattractants also contribute to immune regulation in the lung tumor microenvironment. CCRL2, expressed by gCaps, regulates Natural Killer (NK) cell migration by binding and presenting chemerin to ChemerinR1^+^ NK cells. Upon inflammation, *Ccrl2* expression can also be induced in aCaps (Figure 1B) [19]. CCRL2 deficiency impairs NK cell recruitment and activation, weakening early lung antitumor immune surveillance [19, 59].

Overall, although current evidence remains limited, the spatial and temporal regulation of ACKRs by lung ECs emerges as a critical determinant of effective antitumor immunity. Targeting these receptors to modulate chemokine availability represents a promising strategy to enhance T‐cell‐mediated responses and reshape the tumor microenvironment. Conversely, strategies aimed at increasing CCRL2 expression may support the recruitment of ChemerinR1^+^ NK cells to the lung and further strengthen immune surveillance [19].

This complex and dynamic landscape opens new therapeutic perspectives, which will be briefly discussed in the following section.

### 1.3 Therapeutic Targeting of atypical Receptors for Chemoattractants in Lung Cancer

**FIGURE 3:**
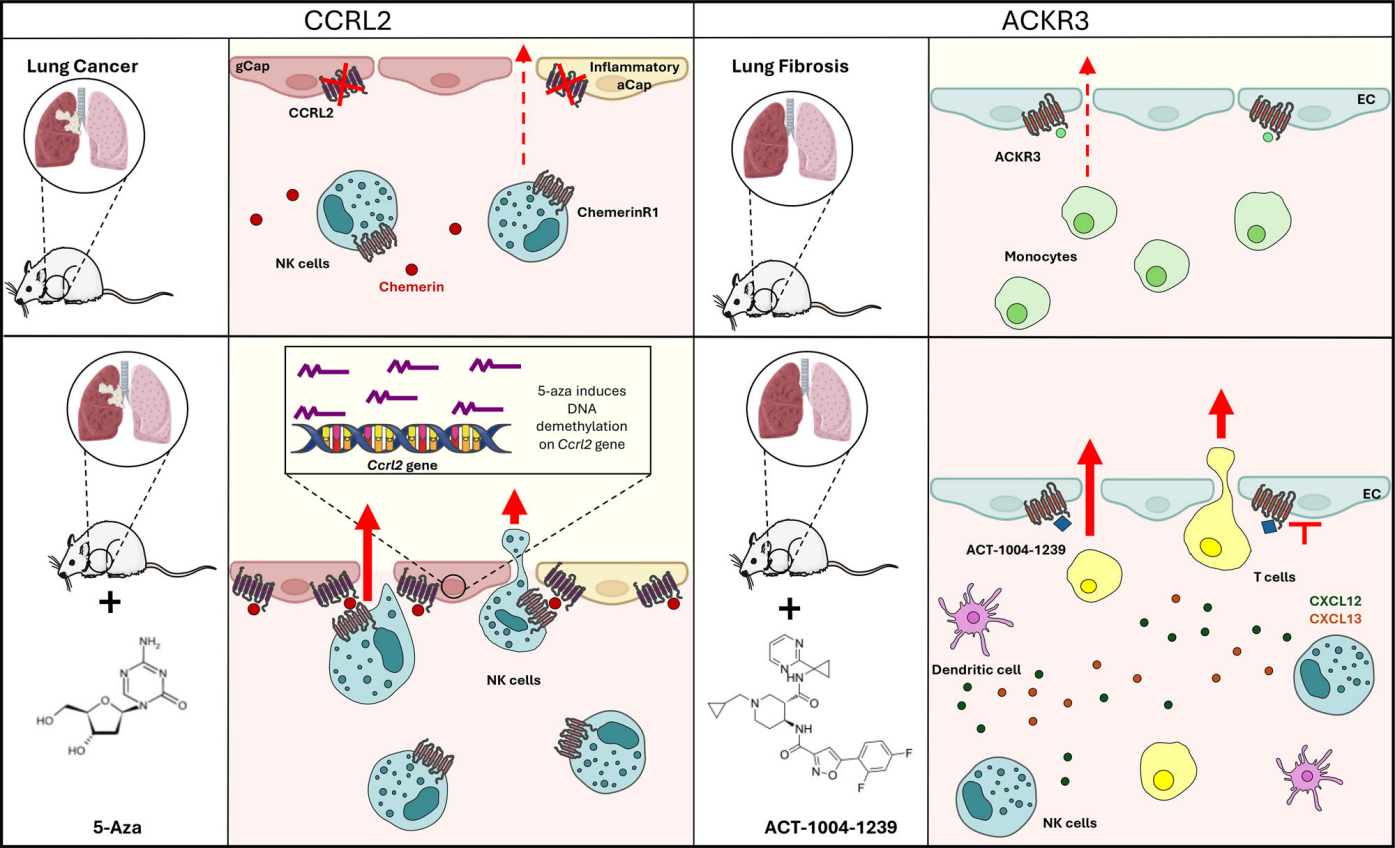
Therapeutic interventions to modulate the atypical chemoattractant receptor expression or function. As CCRL2 expression by the tumor is severely downregulated, treatment with the de‐methylating agent 5‐aza‐2′‐deoxycytidine can restore Ccrl2 protein levels in the lung endothelium, thus promoting lung recruitment of antitumoral ChemerinR1+ NK. ACKR3 acts as a chemokine sink that can modulate the CXCL12/CXCR4 axis, thereby influencing local leukocyte trafficking. The small molecule ACT‐1004‐1239, by blocking ACKR3 binding to CXCL12, can alter the immune cell composition of the lung, thus ameliorating the fibrotic response.

The expression of ACKRs by lung ECs is influenced by the inflammatory status of the tissue, where their expression is associated with the regulation of specific leukocyte subsets. This makes ACKRs attractive candidates for therapeutic intervention, particularly in immunologically “cold” tumors and chronic inflammatory diseases. By fine‐tuning local chemokine levels without directly activating canonical chemokine receptor signaling, ACKRs represent promising pharmacological targets [60, 61] (Figure 3).

Recent work on CCRL2 highlights this potential. CCRL2 expression in lung ECs supports efficient NK homing, yet in human lung cancer it is often downregulated through epigenetic mechanisms [19]. Pharmacological reversal of this repression using hypomethylating agents such as decitabine restores CCRL2 expression, enhances the recruitment of ChemerinR1^+^ NK cells to the lung, and improves tumor control in preclinical models [19]. These findings suggest that modulation of CCRL2 expression may represent a viable strategy to reshape innate immune infiltration and reprogram the tumor microenvironment.

In contrast, direct therapeutic targeting of ACKRs in cancer remains largely unexplored. Limited evidence suggests that ACKR2 expression can be downregulated through activation of the oncogenic signaling pathways, such as the K‐Ras‐B‐Raf‐ERK pathway in Kaposi sarcoma, leading to altered monocyte‐derived macrophage recruitment and polarization toward a pro‐tumoral M2‐like phenotype [62].

More substantial evidence comes from nonmalignant lung diseases, where small molecules targeting ACKR3 have shown therapeutic potential. ACKR3 agonism is a promising approach to attenuate fibrotic processes, although its effects are modest and variable, suggesting that combination approaches may be required [63]. Small molecules, such as ACT‐1004‐1239, reduce lung vascular permeability and leukocyte infiltration in models of acute lung inflammation [44], while the agonist TC14012 limits epithelial damage and fibrosis by modulating profibrotic signaling pathways [43].

Overall, although therapeutic strategies targeting ACKRs are still in their early stages, emerging evidence supports their potential to modulate immune responses and improve disease outcomes in both inflammatory and oncological settings.

### 1.4 Future Perspectives

Overall, the role of atypical chemoattractant receptor expression by ECs in lung immunosurveillance remains incompletely understood, as only a limited number of studies are currently available. A key priority for future research is to better define the spatiotemporal regulation of atypical chemoattractant receptors within the lung vasculature, both in homeostasis and disease, to clarify their contribution to inflammatory responses and their resolution. A major challenge in the field is the limited availability and application of genetic tools that enable selective manipulation of ACKRs in defined endothelial subsets. Recent studies have described conditional mouse models targeting either gCaps or aCaps endothelial cells [21, 64]. However, these models require further validation and characterization before they can be broadly adopted. Nevertheless, the development and application of lung EC‐specific conditional mouse lines, combined with conditional ACKR knockout models, will be instrumental in defining the specific roles of endothelial ACKRs in lung inflammation, fibrosis, and cancer.

In parallel, the possibility of genetically or pharmacologically modulating atypical chemoattractant receptor expression represents an attractive therapeutic avenue. By controlling local chemokine availability, it may be feasible to selectively influence the recruitment and retention of specific immune populations, such as NK or T cells, thereby reshaping the lung immune microenvironment and enhancing protective responses.

Another important aspect that remains largely unexplored is the impact of genetic variants of atypical chemoattractant receptors. Although some variants have been shown to affect chemokine scavenging and receptor interactions [65, 66, 67], their functional relevance to lung endothelial cell pathology is still poorly understood. Similarly, the role of atypical chemoattractant receptors in chemokine and other chemoattractant receptor heterodimerization and biased signaling warrants further investigation in the context of lung EC physiopathology.

Targeting ACKRs in combination with established therapies may offer additional clinical benefits. Modulation of chemokine gradients could potentially improve the responses to immune checkpoint blockade, which currently benefits only a subset of lung cancer patients [68], and may also complement antifibrotic strategies in chronic lung diseases.

Finally, despite the peculiar mechanism of trans‐endothelial migration within the alveolar vasculature, very little is known about its dynamics. Future studies should be devoted to evaluating if trans‐endothelial migration mainly involves a paracellular route, a transcellular route, or both, as well as clarifying the role of both aCap and gCap under homeostatic and inflammatory conditions.

Altogether, a more refined understanding of endothelial cell specialization and compartmentalized atypical chemoattractant receptor expression will be essential to fully elucidate their role in lung immune regulation and to guide the development of novel therapeutic approaches.

## Author Contributions

M.L., M. Thelen, R.B., and S.S. conceived and wrote the manuscript. E.B., F.S., and M. Turchetti contributed to manuscript preparation. G.S. and A.D.P. critically evaluated the manuscript and contributed to the preparation.

## Artificial Intelligence Generated Content

No artificial intelligence‐generated content was utilized.

## Conflicts of Interest

All authors declare no conflicts of interest.

## Data Availability

The authors have nothing to report.

## References

[1] K. Chen , Z. Bao , P. Tang , W. Gong , T. Yoshimura , and J. M. Wang , “Chemokines in Homeostasis and Diseases,” Cellular & Molecular Immunology 15 (2018): 324–334, 10.1038/cmi.2017.134. 29375126 PMC6052829

[2] F. Bachelerie , A. Ben‐Baruch , A. M. Burkhardt , et al., “International Union of Basic and Clinical Pharmacology. LXXXIX. Update on the Extended Family of Chemokine Receptors and Introducing a New Nomenclature for Atypical Chemokine Receptors,” Pharmacological Reviews 66 (2014): 1–79, 10.1124/pr.113.007724. 24218476 PMC3880466

[3] J. W. Griffith , C. L. Sokol , and A. D. Luster , “Chemokines and Chemokine Receptors: Positioning Cells for Host Defense and Immunity,” Annual Review of Immunology 32 (2014): 659–702, 10.1146/annurev-immunol-032713-120145. 24655300

[4] V. Cecchinato , G. D'Agostino , L. Raeli , and M. Uguccioni , “Chemokine Interaction With Synergy‐Inducing Molecules: Fine Tuning Modulation of Cell Trafficking,” Journal of Leukocyte Biology 99 (2016): 851–855, 10.1189/jlb.1MR1015-457R. 26715684 PMC5039040

[5] A. Del Prete , T. Schioppa , L. Tiberio , H. Stabile , and S. Sozzani , “Leukocyte Trafficking in Tumor Microenvironment,” Current Opinion in Pharmacology 35 (2017): 40–47, 10.1016/j.coph.2017.05.004. 28577499

[6] K. Thomas , J. Rossaint , N. Ludwig , et al., “Alveolar Epithelial and Vascular CXCR2 Mediates Transcytosis of CXCL1 in Inflamed Lungs,” Nature Communications 16 (2025): 4846, 10.1038/s41467-025-60174-w. PMC12103508 40413164

[7] S. P. H. Alexander , A. J. Gibb , E. Kelly , et al., “The Concise Guide to PHARMACOLOGY 2025/26: Introduction and Other Protein Targets,” British Journal of Pharmacology 182, no. Suppl 1 (2025): S1–S23, 10.1111/bph.70229. 41461576 PMC13480412

[8] R. Alon , M. Sportiello , S. Kozlovski , et al., “Leukocyte Trafficking to the Lungs and Beyond: Lessons From Influenza for COVID‐19,” Nature Reviews Immunology 21 (2021): 49–64, 10.1038/s41577-020-00470-2. PMC7675406 33214719

[9] J. Rossaint and A. Zarbock , “Tissue‐Specific Neutrophil Recruitment Into the Lung, Liver, and Kidney,” Journal of Innate Immunity 5 (2013): 348–357, 10.1159/000345943. 23257511 PMC6741449

[10] A. Chevigné , D. F. Legler , A. Rot , S. Sozzani , M. Szpakowska , and M. Thelen , “International Union of Basic and Clinical Pharmacology. CXVIII. Update on the Nomenclature for atypical Chemokine Receptors, Including ACKR5,” Pharmacological Reviews 77 (2025): 100012, 10.1124/pharmrev.124.001361. 39952689

[11] I. Comerford and S. R. McColl , “Atypical Chemokine Receptors in the Immune System,” Nature Reviews Immunology 24 (2024): 753–769, 10.1038/s41577-024-01025-5. 38714818

[12] H. G. Lindsay , C. J. Hendrix , J. D. Gonzalez Murcia , C. Haynie , and K. S. Weber , “The Role of Atypical Chemokine Receptors in Neuroinflammation and Neurodegenerative Disorders,” International Journal of Molecular Sciences 24 (2023): 16493, 10.3390/ijms242216493. 38003682 PMC10671188

[13] M. Pruenster and A. Rot , “Throwing Light on DARC,” Biochemical Society Transactions 34 (2006): 1005–1008, 10.1042/BST0341005. 17073738

[14] S. Gonzalvo‐Feo , A. Del Prete , M. Pruenster , et al., “Endothelial Cell–Derived Chemerin Promotes Dendritic Cell Transmigration,” Journal of Immunology 192 (2014): 2366–2373, 10.4049/jimmunol.1302028. 24470498

[15] A. B. Kleist , M. Szpakowska , L. J. Talbot , et al., “Encoding and Decoding Selectivity and Promiscuity in the human Chemokine‐GPCR Interaction Network,” Cell 188 (2025): 3603–3622.e27, 10.1016/j.cell.2025.03.046. 40273912 PMC12435897

[16] M. H. Ulvmar , K. Werth , A. Braun , et al., “The atypical Chemokine Receptor CCRL1 Shapes Functional CCL21 Gradients in Lymph Nodes,” Nature Immunology 15 (2014): 623–630, 10.1038/ni.2889. 24813163

[17] T. Girbl , T. Lenn , L. Perez , et al., “Distinct Compartmentalization of the Chemokines CXCL1 and CXCL2 and the Atypical Receptor ACKR1 Determine Discrete Stages of Neutrophil Diapedesis,” Immunity 49 (2018): 1062–1076.e6, 10.1016/j.immuni.2018.09.018. 30446388 PMC6303217

[18] C. Mazzotti , V. Gagliostro , D. Bosisio , et al., “The Atypical Receptor CCRL2 (C‐C Chemokine Receptor‐Like 2) Does Not Act as a Decoy Receptor in Endothelial Cells,” Frontiers in Immunology 8 (2017): 1233, 10.3389/fimmu.2017.01233. 29056935 PMC5635198

[19] F. Sozio , T. Schioppa , M. Laffranchi , et al., “CCRL2 Expression by Specialized Lung Capillary Endothelial Cells Controls NK‐cell Homing in Lung Cancer,” Cancer Immunology Research 11 (2023): 1280–1295, 10.1158/2326-6066.CIR-22-0951. 37343073

[20] F. Albano , V. Mollica Poeta , L. Zotti , et al., “Selective Expression and Significance of ACKR2 in Lung Aerocytes,” Journal for ImmunoTherapy of Cancer 13 (2025): e009467, 10.1136/jitc-2024-009467. 39843164 PMC11784215

[21] A. Gillich , F. Zhang , C. G. Farmer , et al., “Capillary Cell‐type Specialization in the Alveolus,” Nature 586 (2020): 785–789, 10.1038/s41586-020-2822-7. 33057196 PMC7721049

[22] J. Amersfoort , G. Eelen , and P. Carmeliet , “Immunomodulation by Endothelial Cells — Partnering up With the Immune System?,” Nature Reviews Immunology 22 (2022): 576–588, 10.1038/s41577-022-00694-4. PMC8920067 35288707

[23] S. Daum , L. Decristoforo , M. Mousa , et al., “Unveiling the Immunomodulatory Dance: Endothelial Cells' Function and Their Role in Non‐Small Cell Lung Cancer,” Molecular Cancer 24 (2025): 21, 10.1186/s12943-024-02221-6. 39819502 PMC11737145

[24] J. James , A. Dekan , S. Kacar , et al., “Distinct Populations of Lung Capillary Endothelial Cells and Their Functional Significance,” Communications Biology 9 (2025): 140, 10.1038/s42003-025-09420-x. 41449269 PMC12859004

[25] A. R. Burns , C. W. Smith , and D. C. Walker , “Unique Structural Features That Influence Neutrophil Emigration Into the Lung,” Physiological Reviews 83 (2003): 309–336, 10.1152/physrev.00023.2002. 12663861

[26] L. Zhang , S. Gao , Z. White , Y. Dai , A. B. Malik , and J. Rehman , “Single‐cell Transcriptomic Profiling of Lung Endothelial Cells Identifies Dynamic Inflammatory and Regenerative Subpopulations,” JCI Insight 7 (2022): e158079, 10.1172/jci.insight.158079. 35511435 PMC9220950

[27] J. Oostendorp , M. N. Hylkema , M. Luinge , et al., “Localization and Enhanced mRNA Expression of the Orphan Chemokine Receptor L‐CCR in the Lung in a Murine Model of Ovalbumin‐induced Airway Inflammation,” Journal of Histochemistry & Cytochemistry 52 (2004): 401–410, 10.1177/002215540405200311. 14966207

[28] P. M. Wang , D. L. Kachel , M. F. Cesta , and W. J. Martin , “Direct Leukocyte Migration Across Pulmonary Arterioles and Venules Into the Perivascular Interstitium of Murine Lungs During Bleomycin Injury and Repair,” American Journal of Pathology 178 (2011): 2560–2572, 10.1016/j.ajpath.2011.02.047. 21641381 PMC3124021

[29] L. Sikkema , C. Ramírez‐Suástegui , D. C. Strobl , et al., “An Integrated Cell Atlas of the Lung in Health and Disease,” Nature Medicine 29 (2023): 1563–1577, 10.1038/s41591-023-02327-2. PMC10287567 37291214

[30] C.‐Y. Jiang , L.‐W. Wu , Y.‐W. Liu , et al., “Identification of ACKR4 as an Immune Checkpoint in Pulmonary Arterial Hypertension,” Frontiers in Immunology 14 (2023): 1153573, 10.3389/fimmu.2023.1153573. 37449198 PMC10337759

[31] J. R. Townson and R. J. B. Nibbs , “Characterization of Mouse CCX‐CKR, a Receptor for the Lymphocyte‐attracting Chemokines TECK/mCCL25, SLC/mCCL21 and MIP‐3β/mCCL19: Comparison to human CCX‐CKR,” European Journal of Immunology 32 (2002): 1230, 10.1002/1521-4141(200205)32:5<1230::AID-IMMU1230>3.0.CO;2-L. 11981810

[32] I. Borek , A. Birnhuber , N. F. Voelkel , L. M. Marsh , and G. Kwapiszewska , “The Vascular Perspective on Acute and Chronic Lung Disease,” Journal of Clinical Investigation 133 (2023): e170502, 10.1172/JCI170502. 37581311 PMC10425217

[33] H. Hu , Z. Guo , J. Zhang , et al., “Single‐cell RNA Sequencing Elucidates Potential Mechanisms of Endothelial Cells in Lung Region‐Specific Repair and Remodeling in Combined Pulmonary Fibrosis and Emphysema,” Respiratory Research 27 (2026): 39, 10.1186/s12931-025-03460-x. 41491179 PMC12870540

[34] A. A. Raslan , T. X. Pham , J. Lee , et al., “Lung Injury‐Induced Activated Endothelial Cell States Persist in Aging‐Associated Progressive Fibrosis,” Nature Communications 15 (2024): 5449, 10.1038/s41467-024-49545-x. PMC11211333 38937456

[35] A. Zarbock , J. Bishop , H. Müller , et al., “Chemokine Homeostasis vs. Chemokine Presentation During Severe Acute Lung Injury: The Other Side of the Duffy Antigen Receptor for Chemokines,” American Journal of Physiology‐Lung Cellular and Molecular Physiology 298 (2010): L462–L471, 10.1152/ajplung.00224.2009. 20061440

[36] J. Reutershan , B. Harry , D. Chang , G. J. Bagby , and K. Ley , “DARC on RBC Limits Lung Injury by Balancing Compartmental Distribution of CXC Chemokines,” European Journal of Immunology 39 (2009): 1597–1607, 10.1002/eji.200839089. 19499525 PMC2733952

[37] M. Samus and A. Rot , “Atypical Chemokine Receptors in Cancer,” Cytokine 176 (2024): 156504, 10.1016/j.cyto.2024.156504. 38266462

[38] J. S. Lee , C. W. Frevert , M. M. Wurfel , et al., “Duffy Antigen Facilitates Movement of Chemokine Across the Endothelium in Vitro and Promotes Neutrophil Transmigration in Vitro and in Vivo,” The Journal of Immunology 170 (2003): 5244–5251, 10.4049/jimmunol.170.10.5244. 12734373 PMC4357319

[39] C. A. H. Hansell , S. Love , M. Pingen , G. J. Wilson , M. MacLeod , and G. J. Graham , “Analysis of Lung Stromal Expression of the atypical Chemokine Receptor ACKR2 Reveals Unanticipated Expression in Murine Blood Endothelial Cells,” European Journal of Immunology 50 (2020): 666–675, 10.1002/eji.201948374. 32114694 PMC8638673

[40] R. C. Russo , B. Savino , M. Mirolo , et al., “The atypical Chemokine Receptor ACKR2 Drives Pulmonary Fibrosis by Tuning Influx of CCR2^+^ and CCR5^+^ IFNγ‐producing γδT Cells in Mice,” American Journal of Physiology‐Lung Cellular and Molecular Physiology 314 (2018): L1010–L1025, 10.1152/ajplung.00233.2017. 29469612

[41] L. P. Tavares , C. C. Garcia , A. P. F. Gonçalves , et al., “ACKR2 Contributes to Pulmonary Dysfunction by Shaping CCL5:CCR5‐Dependent Recruitment of Lymphocytes During influenza A Infection in Mice,” American Journal of Physiology‐Lung Cellular and Molecular Physiology 318 (2020): L655–L670, 10.1152/ajplung.00134.2019. 31995405

[42] C. M. Costello , B. McCullagh , K. Howell , et al., “A Role for the CXCL12 Receptor, CXCR7, in the Pathogenesis of Human Pulmonary Vascular Disease,” European Respiratory Journal 39 (2012): 1415–1424, 10.1183/09031936.00044911. 22088972

[43] Z. Cao , R. Lis , M. Ginsberg , et al., “Targeting of the Pulmonary Capillary Vascular Niche Promotes Lung Alveolar Repair and Ameliorates Fibrosis,” Nature Medicine 22 (2016): 154–162, 10.1038/nm.4035. PMC4872630 26779814

[44] L. Pouzol , A. Sassi , N. Baumlin , et al., “CXCR7 Antagonism Reduces Acute Lung Injury Pathogenesis,” Frontiers in Pharmacology 12 (2021): 748740, 10.3389/fphar.2021.748740. 34803691 PMC8602191

[45] C. R. Bastow , M. D. Bunting , E. E. Kara , et al., “Scavenging of Soluble and Immobilized CCL21 by ACKR4 Regulates Peripheral Dendritic Cell Emigration,” Proceedings of the National Academy of Sciences 118 (2021): e2025763118, 10.1073/pnas.2025763118. PMC8092586 33875601

[46] S. Melgrati , O. J. Gerken , M. Artinger , et al., “GPR182 is a Broadly Scavenging atypical Chemokine Receptor Influencing T‐Independent Immunity,” Frontiers in Immunology 14 (2023): 1242531, 10.3389/fimmu.2023.1242531. 37554323 PMC10405735

[47] A. Le Mercier , R. Bonnavion , W. Yu , et al., “GPR182 is an Endothelium‐Specific atypical Chemokine Receptor That Maintains Hematopoietic Stem Cell Homeostasis,” PNAS 118 (2021): e2021596118, 10.1073/pnas.2021596118. 33875597 PMC8092405

[48] F. R. Balkwill , “The Chemokine System and Cancer,” The Journal of Pathology 226 (2012): 148–157, 10.1002/path.3029. 21989643

[49] N. Salazar and B. A. Zabel , “Support of Tumor Endothelial Cells by Chemokine Receptors,” Frontiers in Immunology 10 (2019): 147, [Accessed May 14, 2020] Available at: https://www.frontiersin.org/articles/10.3389/fimmu.2019.00147/full . 30800123 10.3389/fimmu.2019.00147 PMC6375834

[50] R. J. Torphy , E. J. Yee , R. D. Schulick , and Y. Zhu , “Atypical Chemokine Receptors: Emerging Therapeutic Targets in Cancer,” Trends in Pharmacological Sciences 43 (2022): 1085–1097, 10.1016/j.tips.2022.09.009. 36307250 PMC9669249

[51] X. Wang , Z. Wang , Q. Liao , et al., “Spatially Resolved Atlas of Breast Cancer Uncovers Intercellular Machinery of Venular Niche Governing Lymphocyte Extravasation,” Nature Communications 16 (2025): 3348, 10.1038/s41467-025-58511-0. PMC11978809 40199901

[52] S. T. Roach , Q. Wang , R. Patel , et al., “Endothelial ACKR1 Expression Regulates Neutrophil Infiltration and Breast Cancer Metastatic Engraftment in the Lung Metastatic Niche,” *BioRxiv* (2026): 2026:2026.03.15.711832, 10.64898/2026.03.15.711832.

[53] M. Massara , O. Bonavita , A. Mantovani , M. Locati , and R. Bonecchi , “Atypical Chemokine Receptors in Cancer: Friends or Foes?,” Journal of Leukocyte Biology 99 (2016): 927–933, 10.1189/jlb.3MR0915-431RR. 26908826

[54] M. Massara , O. Bonavita , B. Savino , et al., “ACKR2 in Hematopoietic Precursors as a Checkpoint of Neutrophil Release and Anti‐Metastatic Activity,” Nature Communications 9 (2018): 676, 10.1038/s41467-018-03080-8. PMC5813042 29445158

[55] C. A. H. Hansell , A. R. Fraser , A. J. Hayes , et al., “The Atypical Chemokine Receptor Ackr2 Constrains NK Cell Migratory Activity and Promotes Metastasis,” The Journal of Immunology 201 (2018): 2510–2519, 10.4049/jimmunol.1800131. 30158126 PMC6176105

[56] K. E. Quinn , D. I. Mackie , and K. M. Caron , “Emerging Roles of Atypical Chemokine Receptor 3 (ACKR3) in Normal Development and Physiology,” Cytokine 109 (2018): 17–23, 10.1016/j.cyto.2018.02.024. 29903572 PMC6005205

[57] C. E. Whyte , M. Osman , E. E. Kara , et al., “ACKR4 Restrains Antitumor Immunity by Regulating CCL21,” Journal of Experimental Medicine 217 (2020): e20190634, 10.1084/jem.20190634. 32289156 PMC7971131

[58] R. J. Torphy , Y. Sun , R. Lin , et al., “GPR182 limits Antitumor Immunity via Chemokine Scavenging in Mouse Melanoma Models,” Nature Communications 13 (2022): 97, 10.1038/s41467-021-27658-x. PMC8748779 35013216

[59] A. Del Prete , F. Sozio , T. Schioppa , et al., “The Atypical Receptor CCRL2 Is Essential for Lung Cancer Immune Surveillance,” Cancer Immunology Research 7 (2019): 1775–1788, 10.1158/2326-6066.CIR-19-0168. 31484658 PMC7176487

[60] K. S. Crawford and B. F. Volkman , “Prospects for Targeting ACKR1 in Cancer and Other Diseases,” Frontiers in Immunology 14 (2023): 1111960, 10.3389/fimmu.2023.1111960. 37006247 PMC10050359

[61] C. N. Moscinski , C. Born , and D. S. Heffernan , “The Role of Duffy Antigen Receptor for Chemokines in Acute Respiratory Distress Syndrome,” Journal of Leukocyte Biology 118 (2026): qiag036, 10.1093/jleuko/qiag036. 41840847

[62] B. Savino , N. Caronni , A. Anselmo , et al., “ERK‐dependent Downregulation of the atypical Chemokine Receptor D6 Drives Tumor Aggressiveness in Kaposi Sarcoma,” Cancer Immunology Research 2 (2014): 679–689, 10.1158/2326-6066.CIR-13-0202. 24844911

[63] T. Van Loy , S. De Jonghe , K. Castermans , et al., “Stimulation of the Atypical Chemokine Receptor 3 (ACKR3) by a Small‐Molecule Agonist Attenuates Fibrosis in a Preclinical Liver but Not Lung Injury Model,” Cellular and Molecular Life Sciences 79 (2022): 293, 10.1007/s00018-022-04317-y. 35562519 PMC9106635

[64] Z. Zhang and B. Zhou , “Generation of *Plvap‐CreER* and *Car4‐CreER* for Genetic Targeting of Distinct Lung Capillary Populations,” Journal of Genetics and Genomics 49 (2022): 1093–1100, 10.1016/j.jgg.2022.08.001. 36028133

[65] J. D. G. Murcia , A. Weinert , C. M. T. Freitas , et al., “Atypical Chemokine Receptor ACKR2‐V41A Has Decreased CCL2 Binding, Scavenging, and Activation, Supporting Sustained Inflammation and Increased Alzheimer's Disease Risk,” Scientific Reports 10 (2020): 8019, 10.1038/s41598-020-64755-1. 32415244 PMC7229167

[66] M. Laffranchi , E. M. Paraboschi , F. Bianchetto‐Aguilera , et al., “Neutrophils Restricted Contribution of CCRL2 Genetic Variants to COVID‐19 Severity,” Heliyon 11 (2025): e41267, 10.1016/j.heliyon.2024.e41267. 39811276 PMC11731188

[67] P. An , R. Li , J. M. Wang , et al., “Role of Exonic Variation in Chemokine Receptor Genes on AIDS: CCRL2 F167Y Association With Pneumocystis Pneumonia,” PLoS Genetics 7 (2011): e1002328, 10.1371/journal.pgen.1002328. 22046140 PMC3203199

[68] E. B. Garon , M. D. Hellmann , N. A. Rizvi , et al., “Five‐Year Overall Survival for Patients with Advanced Non‒Small‐Cell Lung Cancer Treated with Pembrolizumab: Results from the Phase I KEYNOTE‐001 Study,” Journal of Clinical Oncology 37 (2019): 2518–2527, 10.1200/JCO.19.00934. 31154919 PMC6768611

[69] M. C. Szabo , K. S. Soo , A. Zlotnik , and T. J. Schall , “Chemokine Class Differences in Binding to the Duffy Antigen‐Erythrocyte Chemokine Receptor,” Journal of Biological Chemistry 270 (1995): 25348–25351, 10.1074/jbc.270.43.25348. 7592697

[70] L. Gardner , A. M. Patterson , B. A. Ashton , M. A. Stone , and J. Middleton , “The Human Duffy Antigen Binds Selected Inflammatory but Not Homeostatic Chemokines,” Biochemical and Biophysical Research Communications 321 (2004): 306–312, 10.1016/j.bbrc.2004.06.146. 15358176

[71] J. C. Gutjahr , K. S. Crawford , D. R. Jensen , et al., “The Dimeric Form of CXCL12 Binds to Atypical Chemokine Receptor 1,” Science Signaling 14 (2021): eabc9012, 10.1126/scisignal.abc9012. 34404752 PMC9015690

[72] T. R. L. Klei , F. Aglialoro , F. P. J. Mul , et al., “Differential Interaction Between DARC and SDF‐1 on Erythrocytes and Their Precursors,” Scientific Reports 9 (2019): 16245, 10.1038/s41598-019-52186-6. 31700087 PMC6838059

[73] A. N. Spaan , T. Reyes‐Robles , C. Badiou , et al., “ *Staphylococcus aureus* Targets the Duffy Antigen Receptor for Chemokines (DARC) to Lyse Erythrocytes,” Cell Host & Microbe 18 (2015): 363–370, 10.1016/j.chom.2015.08.001. 26320997 PMC4578157

[74] M. J. Bolton and R. F. Garry , “Sequence Similarity Between the Erythrocyte Binding Domain 1 of the Plasmodium Vivax Duffy Binding Protein and the V3 Loop of HIV‐1 Strain MN Reveals Binding Residues for the Duffy Antigen Receptor for Chemokines,” Virology Journal 8 (2011): 45, 10.1186/1743-422X-8-45. 21281498 PMC3040140

[75] C. E. Chitnis and L. H. Miller , “Identification of the Erythrocyte Binding Domains of Plasmodium Vivax and Plasmodium Knowlesi Proteins Involved in Erythrocyte Invasion,” The Journal of Experimental Medicine 180 (1994): 497–506, 10.1084/jem.180.2.497. 8046329 PMC2191600

[76] R. Luís , B. F. Volkman , M. Szpakowska , and A. Chevigné , “Extended Repertoire of CXC Chemokines Acting as Agonists and Antagonists of the Human and Murine Atypical Chemokine Receptor ACKR2,” Journal of Leukocyte Biology 117 (2025): qiaf013, 10.1093/jleuko/qiaf013. 39903605 PMC12017343

[77] A. Chevigné , B. Janji , M. Meyrath , et al., “CXCL10 Is an Agonist of the CC Family Chemokine Scavenger Receptor ACKR2/D6,” Cancers 13 (2021): 1054, 10.3390/cancers13051054. 33801414 PMC7958614

[78] R. J. Nibbs , S. M. Wylie , J. Yang , N. R. Landau , and G. J. Graham , “Cloning and Characterization of a Novel Promiscuous Human β‐Chemokine Receptor D6,” Journal of Biological Chemistry 272 (1997): 32078–32083, 10.1074/jbc.272.51.32078. 9405404

[79] R. J. Nibbs , S. M. Wylie , I. B. Pragnell , and G. J. Graham , “Cloning and Characterization of a Novel Murine β Chemokine Receptor, D6,” Journal of Biological Chemistry 272 (1997): 12495–12504, 10.1074/jbc.272.19.12495. 9139699

[80] R. J. Nibbs , J. Yang , N. R. Landau , J. H. Mao , and G. J. Graham , “LD78β, A Non‐Allelic Variant of Human MIP‐1α (LD78α), Has Enhanced Receptor Interactions and Potent HIV Suppressive Activity,” Journal of Biological Chemistry 274 (1999): 17478–17483, 10.1074/jbc.274.25.17478. 10364178

[81] M. V. Goncharuk , D. Roy , M. A. Dubinnyi , et al., “Purification of Native CCL7 and Its Functional Interaction With Selected Chemokine Receptors,” Protein Expression and Purification 171 (2020): 105617, 10.1016/j.pep.2020.105617. 32145391 PMC7212033

[82] L. B. Ford , V. Cerovic , S. W. F. Milling , G. J. Graham , C. A. H. Hansell , and R. J. B. Nibbs , “Characterization of Conventional and Atypical Receptors for the Chemokine CCL2 on Mouse Leukocytes,” Journal of Immunology 193 (2014): 400–411, 10.4049/jimmunol.1303236. PMC4065784 24890717

[83] R. Bonecchi , M. Locati , E. Galliera , et al., “Differential Recognition and Scavenging of Native and Truncated Macrophage‐Derived Chemokine (Macrophage‐Derived Chemokine/CC Chemokine Ligand 22) by the D6 Decoy Receptor,” The Journal of Immunology 172 (2004): 4972–4976, 10.4049/jimmunol.172.8.4972. 15067078

[84] K. D. Hewit , A. Fraser , R. J. B. Nibbs , and G. J. Graham , “The N‐Terminal Region of the Atypical Chemokine Receptor ACKR2 Is a Key Determinant of Ligand Binding,” Journal of Biological Chemistry 289 (2014): 12330–12342, 10.1074/jbc.M113.534545. 24644289 PMC4007430

[85] J. M. Burns , B. C. Summers , Y. Wang , et al., “A Novel Chemokine Receptor for SDF‐1 and I‐TAC Involved in Cell Survival, Cell Adhesion, and Tumor Development,” The Journal of Experimental Medicine 203 (2006): 2201–2213, 10.1084/jem.20052144. 16940167 PMC2118398

[86] M. Szpakowska , N. Dupuis , A. Baragli , et al., “Human Herpesvirus 8‐Encoded Chemokine vCCL2/vMIP‐II Is an Agonist of the atypical Chemokine Receptor ACKR3/CXCR7,” Biochemical Pharmacology 114 (2016): 14–21, 10.1016/j.bcp.2016.05.012. 27238288

[87] S. Alampour‐Rajabi , O. El Bounkari , A. Rot , et al., “MIF Interacts With CXCR7 to Promote Receptor Internalization, ERK1/2 and ZAP‐70 Signaling, and Lymphocyte Chemotaxis,” FASEB Journal 29 (2015): 4497–4511, 10.1096/fj.15-273904. 26139098

[88] Y. Ikeda , H. Kumagai , A. Skach , M. Sato , and M. Yanagisawa , “Modulation of Circadian Glucocorticoid Oscillation via Adrenal Opioid‐CXCR7 Signaling Alters Emotional Behavior,” Cell 155 (2013): 1323–1336, 10.1016/j.cell.2013.10.052. 24315101 PMC3934808

[89] M. Szpakowska , A. M. Decker , M. Meyrath , et al., “The Natural Analgesic Conolidine Targets the Newly Identified Opioid Scavenger ACKR3/CXCR7,” Signal Transduction and Targeted Therapy 6 (2021): 209, 10.1038/s41392-021-00548-w. 34075018 PMC8169647

[90] M. Meyrath , M. Szpakowska , J. Zeiner , et al., “The Atypical Chemokine Receptor ACKR3/CXCR7 Is a Broad‐Spectrum Scavenger for Opioid Peptides,” Nature Communications 11 (2020): 3033, 10.1038/s41467-020-16664-0. PMC7305236 32561830

[91] J. Gosling , D. J. Dairaghi , Y. Wang , et al., “Cutting Edge: Identification of a Novel Chemokine Receptor That Binds Dendritic Cell‐ and T Cell‐Active Chemokines Including ELC, SLC, and TECK,” The Journal of Immunology 164 (2000): 2851–2856, 10.4049/jimmunol.164.6.2851. 10706668

[92] M. Meyrath , N. Reynders , T. Uchański , A. Chevigné , and M. Szpakowska , “Systematic Reassessment of Chemokine‐Receptor Pairings Confirms CCL20 but Not CXCL13 and Extends the Spectrum of ACKR4 Agonists to CCL22,” Journal of Leukocyte Biology 109 (2021): 373–376, 10.1002/JLB.2AB0520-275R. 32480426

[93] C. Matti , G. D'Uonnolo , M. Artinger , et al., “CCL20 is a Novel Ligand for the Scavenging atypical Chemokine Receptor 4,” Journal of Leukocyte Biology 107 (2020): 1137–1154, 10.1002/JLB.2MA0420-295RRR. 32533638

[94] Z. Sun , R. J. Torphy , E. N. Miller , et al., “GPR182 is a Lipoprotein Receptor for Dietary Fat Absorption,” Journal of Clinical Investigation 136 (2026): e200857, 10.1172/JCI200857. 41874575 PMC13262718

[95] R. Bonnavion , S. Liu , H. Kawase , K. A. Roquid , and S. Offermanns , “Large Chemokine Binding Spectrum of Human and Mouse Atypical Chemokine Receptor GPR182 (ACKR5),” Frontiers in Pharmacology 14 (2023): 1297596, 10.3389/fphar.2023.1297596. 38026988 PMC10646305

[96] O. De Henau , G.‐N. Degroot , V. Imbault , et al., “Signaling Properties of Chemerin Receptors CMKLR1, GPR1 and CCRL2,” PLoS ONE 11 (2016): e0164179, 10.1371/journal.pone.0164179. 27716822 PMC5055294

[97] Z. Su , J. Brooks , J. Pelker , et al., “Studies With Neutralizing Antibodies Suggest CXCL8‐Mediated Neutrophil Activation Is Independent of C‐C Motif Chemokine Receptor‐Like 2 (CCRL2) Ligand Binding Function,” PLoS ONE 18 (2023): e0280590, 10.1371/journal.pone.0280590. 36662882 PMC9858354

[98] Y. Hao , T. Stuart , M. H. Kowalski , et al., “Dictionary Learning for Integrative, Multimodal and Scalable Single‐Cell Analysis,” Nature Biotechnology 42 (2024): 293–304, 10.1038/s41587-023-01767-y. PMC10928517 37231261

